# Goals, dreams and deadlines

**DOI:** 10.4103/0019-5545.39748

**Published:** 2008

**Authors:** T. S. Sathyanarayana Rao

**Affiliations:** Department of Psychiatry, JSS Medical College Hospital, Mysore - 570 004, India

I am happy to present the first issue on the silver jubilee of the journal on the New Year. The office is to a great extent stabilized and true to the spirit expressed by the past editor Dr. G. Swaminath, the handing over of the charge was smooth and serene. Since taking over, it is again running after to cover the big backlog, securing budget and meeting deadlines. It is Paul Hanna[1] who said “Goals are dreams with deadlines”! With apologies to her, the title is a short version of it. Apart from all the help received from the office bearers, EC members, reviewers and the publisher which I acknowledge, I also appreciate the tremendous load of work shared by the Editorial team of the Journal.

Our website is doing well. The major changes have been introduced for the current year concerning many aspects of the review process, simplification of submission and tracking of the articles and host of appliances for editorial administrators and reviewers at *www.journalonweb.com/psychiatry*

The commemorative volume has evoked a very positive response all through. The budget was the major constraint in the dispatch of the commemorative volume and the current issue. Somehow pharmaceutical companies have chipped in their contribution. I think the provision for adequate budgetary support should be made for the smooth and timely functioning and hope that the esteemed EC will look into the matter.

As for the editorial processing is concerned there has been some delay and it is because of the backlog that was built up due to the turmoil in the society in the past year. I personally apologise for whatever reasons the delay was in the past and promise that the process will be hastened in the coming weeks. Even under tremendous pressure, the current issue was made available on time at our website on 31st March 2008 and there are provisions made to announce the forthcoming topics and highlights. A special symposium on dementia coordinated by Dr. K. S. Shaji and another one on neuroplasticity are being planned for this year. There are many queries by some of the authors who have submitted the articles in 2004-06 and who have not received communication so far may please contact the editor for the update and uploading. The delay may be purely technical and we need to work together.

We had sent the mailers earlier for the members willing to be on the reviewer's panel asking for the details. Many have not yet responded. I earnestly request the members to send their details, phone and e-mail addresses: so also the areas of interest and specialization. You can directly upload your details at *http://www.indianjpsychiatry.org/joinus.asp* which is preferable or to my email *editor@indianjpsychiatry.org* or my personal E-mail ID *tssrao19@yahoo.com*. I look forward to your association.

The non-members subscriptions involved a great deal of clerical work and were consistent source of enquiries. To streamline the process the whole work has been out-sourced to medknow publications from this year. I am confident the professional approach will be appreciated by our esteemed non-member subscribers.

We have included an additional column PERISCOPE from this issue which will be attended by our assistant editor, Dr. G. Swaminath regularly. A CME on each issue is being introduced which will be covered by Dr. Chittaranjan Andrade in this and on coming issues. As you have seen in these pages there are many articles emphasizing psychological aspects, stigma and disabilities. The shift is a conscious one and you are going to see many such changes. Infact, we have lined up a series of 3 articles by Dr. C. Shamasundar on psychological aspects in the section on cultural psychiatry, starting from next issue.

## Ultimately to quote what Samuel Johnson[2] said:

‘Knowledge is of two kinds. We know a subject ourselves, or we know where we can find information on it’. I would like to see our IJP in the forefront of this knowledge movement concerning sychiatry and behavioral sciences. I wish you a hay reading.
